# Diagnostic Challenges of Regional Cardiac Tamponade in a Severely Volume-Overloaded Dialysis Patient: The Role of Multimodality Imaging

**DOI:** 10.7759/cureus.117064

**Published:** 2026-09-28

**Authors:** Alireza Izadian Bidgoli, Alberto Gomez Veliz, Angela C Gallagher, Alberto Gomez Usatorres, Hellen Ailette Gomez Usatorres

**Affiliations:** 1 Internal Medicine, American University of the Caribbean School of Medicine, Miami, USA; 2 Internal Medicine, Jackson Memorial Hospital, Miami, USA; 3 Internal Medicine, Universidad Hispanoamericana, San José, CRI

**Keywords:** cardiac surgery, hemodialysis, loculated pericardial effusion, multimodality imaging, regional cardiac tamponade

## Abstract

Localized postoperative cardiac tamponade is an uncommon clinical-hemodynamic syndrome that may occur without the classic findings of circumferential tamponade. We present a 22-year-old man with dialysis-dependent kidney failure and multiple recent cardiothoracic operations who was admitted because persistent sinus tachycardia prevented completion of hemodialysis. Despite profound total-body volume overload manifested by anasarca, pulmonary edema, ascites, and recurrent pleural effusions, he developed progressive hypotension, respiratory compromise, and increasing vasopressor requirements. Computed tomography demonstrated an enlarging loculated pericardial collection. Transthoracic echocardiography showed right ventricular compression and a dilated, non-collapsing inferior vena cava, raising concern for obstructive physiology.

Intraoperative transesophageal echocardiography did not demonstrate classic global tamponade findings but identified an organized pericardial collection causing focal right atrial impingement. The patient’s clinical deterioration, combined with the multimodality imaging findings, supported a diagnosis of clinically significant regional postoperative tamponade. Because the organized collection and extensive adhesions were unlikely to be adequately treated by percutaneous drainage, he underwent urgent surgical decompression through a repeat pericardial window. Approximately 680 mL was drained from the pericardial and right pleural spaces combined.

Following decompression and controlled renal replacement therapy, vasopressor requirements, tachycardia, respiratory status, and volume overload improved. This case demonstrates that profound total-body volume overload does not exclude regional cardiac tamponade and emphasizes that echocardiographic findings must be interpreted within the overall clinical and hemodynamic context.

## Introduction

Cardiac tamponade is a clinical-hemodynamic syndrome caused by impaired cardiac filling from elevated intrapericardial pressure [1]. Echocardiography and computed tomography (CT) can demonstrate pericardial collections, chamber compression, and altered flow; however, imaging findings alone neither establish nor exclude the diagnosis [1-3]. Clinical and echocardiographic manifestations vary according to the rate and distribution of fluid accumulation, pericardial compliance, and intracardiac filling pressures [1,2,4].

After cardiac surgery, adhesions and organized blood or fluid may create loculated collections that selectively compress individual chambers, producing regional tamponade without the classic features of circumferential tamponade [2,3,5]. Gopalakrishnan and Pillai described a localized postoperative effusion causing right ventricular diastolic free-wall collapse that resolved after drainage [5], whereas other reports have described isolated left-sided compression [4]. Because the available literature consists predominantly of case reports and small series, the frequency of specific regional chamber-collapse patterns remains uncertain [2-5]. Postoperative anatomy, limited acoustic windows, organized material, and changing loading conditions may also produce discordant findings across imaging modalities [2,3].

This case is notable because regional obstructive physiology developed despite profound total-body volume overload in a dialysis-dependent patient with multiple recent cardiac operations and recurrence after prior surgical drainage. Transthoracic and intraoperative transesophageal echocardiography produced incomplete and discordant findings, while the overall clinical deterioration and multimodality imaging supported urgent surgical decompression. The case demonstrates that severe systemic volume overload does not exclude regional tamponade and that imaging must be interpreted within the patient’s clinical and hemodynamic context [1-4].

## Case presentation

Clinical presentation

A 22-year-old man with congenital posterior urethral valves underwent deceased-donor kidney transplantation in 2015. His post-transplant course was complicated by recurrent urinary tract infections, acute kidney injury, and T-cell-mediated rejection, resulting in renal allograft failure and resumption of intermittent hemodialysis in 2022. His additional comorbidities included anemia and mineral and bone disorder associated with chronic kidney disease, hypothyroidism, hypoalbuminemia, and recurrent volume overload. Cardiac evaluation preceding his initial valve operation documented normal left ventricular systolic function.

His recent cardiac history included *Staphylococcus lugdunensis* aortic valve endocarditis complicated by an aortic root abscess and a membranous ventricular septal defect. Fifty-three days before the index admission, he underwent aortic root abscess debridement, bioprosthetic aortic valve replacement, and patch repair of the septal defect during the same operation. He was discharged 10 days later. Before the subsequent cardiac arrest, he required an intervening hospitalization for symptomatic bilateral pleural effusions, which were treated with pleural drainage and chemical pleurodesis, followed by discharge.

Twenty days before the index admission, the patient sustained a pulseless electrical activity arrest during outpatient hemodialysis and was initially treated at an outside hospital. The outside hospital’s resuscitation records were unavailable for review; therefore, arrest downtime and the duration of resuscitation before return of spontaneous circulation could not be verified. Five days later, he was transferred to our institution while intubated, sedated, and receiving vasopressor support. Subsequent evaluation identified a persistent ventricular septal defect with left-to-right shunting.

Twelve days before the index admission, he underwent a separate redo operation comprising repeat bioprosthetic aortic valve replacement, aortic root debridement and reconstruction, patch closure of the persistent ventricular septal defect and an associated Gerbode defect, and ascending aortic replacement. The chest was initially left open, followed by mediastinal washout and chest closure the following day. He was discharged home with home health care 10 days after the redo operation, with arrangements for continued antimicrobial treatment and outpatient hemodialysis. At discharge, he was hemodynamically stable, breathing room air, and had no documented focal neurologic deficits.

The index hospitalization (Day 1) began two days after discharge and 12 days after the redo operation, when the patient was readmitted from his community outpatient hemodialysis center because persistent sinus tachycardia prevented completion of dialysis. During this admission, recurrent hemorrhagic bilateral pleural effusions and hemothoraces required thoracenteses, bilateral chest-tube placement on Day 8, and intrapleural fibrinolytic therapy. These chest tubes were subsequently removed. On Day 23, he underwent a left mini-thoracotomy with extensive adhesiolysis, evacuation of a large postoperative pericardial collection, and placement of pericardial and thoracostomy drains. The pericardial drain was removed on Day 25. A left pleural pigtail catheter was subsequently placed on Day 35 for a persistent anterior loculated hydropneumothorax.

Despite these interventions, pleural and pericardial fluid reaccumulated in the setting of severe anasarca and dialysis-dependent kidney failure. On Day 36, persistent tachycardia prompted escalation to intermediate-level care. By Day 41, his heart rate repeatedly reached 120-150 beats/min, hypotension had worsened, and vasopressor support and supplemental oxygen were required. This clinical deterioration, together with the recurrent loculated pericardial collection, raised concern for regional cardiac compression contributing to obstructive shock. Table 1 summarizes the major clinical events and interventions.

**Table 1:** Timeline of major clinical events and interventions. Day 1 denotes the index admission for persistent sinus tachycardia that prevented completion of outpatient hemodialysis. Earlier events are expressed relative to this admission, and the index hospitalization extends through discharge on Day 54. Approximate intervals are used where exact timing was unavailable. Records of resuscitation at the outside hospital were unavailable, preventing verification of arrest downtime and resuscitation duration. Abbreviations: AVR: aortic valve replacement; VSD: ventricular septal defect; PEA: pulseless electrical activity; CRRT: continuous renal replacement therapy

Relative timeframe	Clinical event or intervention
53 days before admission	Aortic root abscess debridement, initial bioprosthetic AVR, and patch repair of a membranous VSD during the same operation.
43 days before admission	Discharge following the initial cardiac operation.
Approximately 3-6 weeks before admission	Hospitalization for symptomatic bilateral pleural effusions, treated with pleural drainage and chemical pleurodesis, followed by discharge.
20 days before admission	PEA arrest during outpatient hemodialysis, followed by treatment at an outside hospital.
15 days before admission	Transfer to the reporting institution while intubated, sedated, and receiving vasopressor support; subsequent evaluation identified a persistent VSD with left-to-right shunting.
12 days before admission	Redo bioprosthetic AVR, aortic root debridement and reconstruction, patch closure of the persistent VSD and associated Gerbode defect, and ascending aortic replacement.
11 days before admission	Mediastinal washout and chest closure following the redo operation.
2 days before admission	Discharged home with home health care, continued antimicrobial treatment, and outpatient hemodialysis.
Day 1	Index readmission from outpatient hemodialysis because persistent sinus tachycardia prevented completion of treatment.
Day 8	Bilateral chest-tube placement for recurrent pleural collections.
Between Days 8 and 23	Removal of the initial bilateral chest tubes.
Day 23	Left mini-thoracotomy, extensive adhesiolysis, surgical evacuation of the pericardial collection, and placement of pericardial and thoracostomy drains.
Day 25	Removal of the pericardial drain.
Day 35	Left pleural pigtail catheter placement for a persistent anterior loculated hydropneumothorax.
Day 36	Escalation to intermediate-level care for persistent tachycardia.
Day 41	Progressive hypotension and respiratory deterioration requiring vasopressor support; urgent right mini-thoracotomy and repeat pericardial window, with approximately 680 mL evacuated from the pericardial and right pleural spaces combined.
Day 41-49	CRRT following surgical decompression for controlled volume removal during hemodynamic recovery.
Day 50	Resumption of intermittent hemodialysis after discontinuation of CRRT.
Day 54	Discharge home with continued outpatient hemodialysis and planned cardiothoracic follow-up.

Initial assessment and physical exam

At the time of acute deterioration on Day 41, the patient was chronically ill-appearing but awake and initially able to follow commands. His heart rate was approximately 113 beats/min, with documented excursions above 150 beats/min. His blood pressure was approximately 95/65 mmHg, with intermittent decreases in systolic pressure to approximately 80 mmHg, and his respiratory rate was 24 breaths/min. He required escalating supplemental oxygen, including high-flow nasal oxygen at 20 L/min with a fraction of inspired oxygen of 0.60.

Because of worsening hypotension, norepinephrine was initiated at the documented rate of 5 μg/min. A contemporaneous body weight at the time of vasopressor initiation could not be reliably verified. Given the patient’s severe anasarca and substantial subsequent fluid removal with dialysis and continuous renal replacement therapy, conversion using a weight recorded later in the hospitalization would not have provided a reliable weight-normalized dose.

Cardiovascular examination demonstrated persistent sinus tachycardia without a prominent murmur. Jugular venous distension was not readily apparent. Pulmonary examination revealed findings consistent with pulmonary edema and complex bilateral pleural disease, and a left-sided pigtail catheter remained in place for a loculated pleural collection. The abdomen was tense and distended, with clinically evident ascites. Diffuse pitting edema and anasarca were present. The patient remained alert without a persistent focal neurologic deficit. His surgical incisions were intact, and no overt external source of infection was identified.

Laboratory and diagnostic testing

Laboratory evaluation near the onset of hemodynamic deterioration demonstrated a serum sodium concentration of 135 mmol/L, potassium of 5.5 mmol/L, bicarbonate of 29 mmol/L, blood urea nitrogen of 32 mg/dL, and creatinine of 4.08 mg/dL in the setting of dialysis-dependent kidney failure. The white blood cell count was 4.5 × 10⁹/L, hemoglobin was 7.6 g/dL, hematocrit was 26.1%, and platelet count was 164 × 10⁹/L. Prothrombin time was 18.4 seconds, international normalized ratio was 1.51, and activated partial thromboplastin time was mildly prolonged. Serum albumin remained reduced, generally between 2.7 and 3.0 g/dL, consistent with his severe anasarca and chronic illness. The N-terminal pro-B-type natriuretic peptide concentration was markedly elevated at approximately 29,200 pg/mL. Venous blood gas analysis showed a pH of 7.40, partial pressure of carbon dioxide of approximately 48-50 mmHg, and bicarbonate of approximately 29-30 mmol/L. Serum lactate was not substantially elevated (Table 2).

**Table 2:** Laboratory findings at admission, during hospitalization, and at discharge. Values in the “During hospitalization” column correspond to the period of progressive tachycardia and hypotension surrounding evaluation and surgical drainage of the recurrent pericardial collection. Reference intervals represent standard adult laboratory ranges. Arrows indicate values above or below the reference range, and "-" indicates unavailable measurements. NT-proBNP values should be interpreted cautiously in the setting of dialysis-dependent kidney failure. Abbreviations: WBC: white blood cell count; BUN: blood urea nitrogen; AST: aspartate aminotransferase; ALT: alanine aminotransferase; PT: prothrombin time; INR: international normalized ratio; aPTT: activated partial thromboplastin time; NT-proBNP: N-terminal pro-B-type natriuretic peptide; g/dL: grams per deciliter; mg/dL: milligrams per deciliter; mmol/L: millimoles per liter; pg/mL: picograms per milliliter; U/L: units per liter.

Laboratory parameter	Admission	During hospitalization	Discharge	Reference range	Unit
White blood cell count	4.6	4.5	8.2	4.0-11.0	×10⁹/L
Hemoglobin	6.3	7.6	9.8	13.5-17.5	g/dL
Hematocrit	20.2	26.1	32.8	41-53	%
Platelet count	161	164	235	150-400	×10⁹/L
Sodium	138	135	134	135-145	mmol/L
Potassium	5.5	4.3	4.4	3.5-5.1	mmol/L
Chloride	104	98	95	98-107	mmol/L
Bicarbonate	20	29	28	22-29	mmol/L
Blood urea nitrogen	60	32	29	7-20	mg/dL
Creatinine	9.86	4.08	4.50	0.7-1.3	mg/dL
Calcium	9.3	9.1	10.3	8.6-10.2	mg/dL
Magnesium	2.1	1.9	2.2	1.7-2.4	mg/dL
Phosphorus	6.8	3.9	4.6	2.5-4.5	mg/dL
Total protein	4.9	5.7	5.9	6.4-8.3	g/dL
Albumin	2.7	3.0	3.7	3.5-5.0	g/dL
Total bilirubin	1.1	0.5	0.6	0.2-1.2	mg/dL
Aspartate aminotransferase	63	20	19	10-40	U/L
Alanine aminotransferase	17	4	7	7-56	U/L
Alkaline phosphatase	140 ↑	135	120	40-129	U/L
Prothrombin time	-	18.4	14.7	11.5-14.5	seconds
International normalized ratio	-	1.51	1.14	0.8-1.2	-
Activated partial thromboplastin time	-	40	29	25-35	seconds
NT-proBNP	21,000	29,200	-	<125	pg/mL

Serial blood and body-fluid cultures obtained during the later hospitalization showed no growth. The patient remained afebrile and did not demonstrate leukocytosis at the time of hemodynamic deterioration, making uncontrolled bloodstream infection a less compelling explanation for the acute shock state. Ascitic fluid contained relatively few nucleated cells and was culture-negative. Chronic anemia was attributed to kidney disease, repeated surgical procedures, and blood loss into the pleural and pericardial spaces and was managed with red blood cell transfusions, erythropoiesis-stimulating therapy, and iron supplementation.

CT of the chest demonstrated a complex multiloculated left anterior hydropneumothorax (Figure 1A), a hyperattenuating left lower and subpulmonic pleural collection compatible with blood products or organizing clot (Figure 1B), and a multiloculated right pleural effusion (Figure 1C). The examination also demonstrated interval enlargement of a moderate pericardial effusion, with a prominent loculated component inferior to the right ventricle measuring approximately 8.4 × 6.0 cm. Generalized anasarca and progressive ascites were also present.

**Figure 1:**
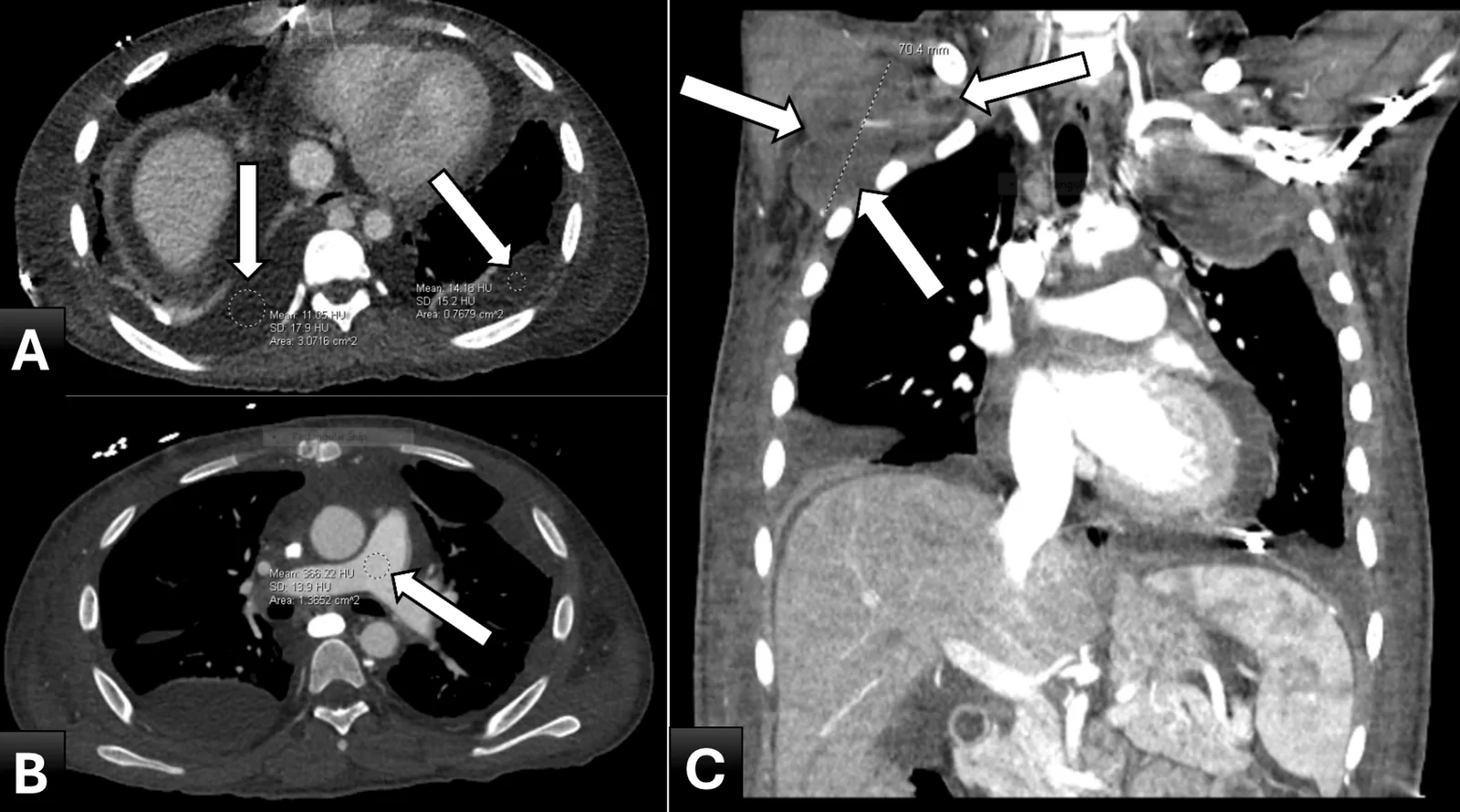
Computed tomography (CT) findings of complex bilateral postoperative pleural disease. (A) Axial chest CT demonstrates a multiloculated left anterior hydropneumothorax, with pleural air and fluid components. (B) Axial chest CT demonstrates a hyperattenuating left lower and subpulmonic pleural collection compatible with blood products or organizing clot. (C) Coronal chest CT demonstrates a multiloculated right pleural effusion extending into the apical pleural space. White arrows identify the corresponding abnormalities.

A technically difficult transthoracic echocardiogram obtained during the acute deterioration demonstrated grossly preserved left ventricular systolic function. The right ventricle was normal in size but had mildly to moderately reduced systolic function. Additional findings included mild mitral regurgitation, moderate-to-severe tricuspid regurgitation, a dilated inferior vena cava without normal inspiratory collapse, and a large pericardial effusion located predominantly anterior to the right ventricle. Color Doppler imaging demonstrated a broad regurgitant jet across the tricuspid valve, consistent with moderate-to-severe tricuspid regurgitation (Figure 2). The collection produced right ventricular compression. Respiratory variation measured approximately 23% across the mitral inflow and 35% across the tricuspid inflow. In conjunction with inferior vena cava plethora and the clinical shock state, these findings were considered suggestive of cardiac tamponade. Representative color and spectral Doppler images obtained during the hemodynamic deterioration are shown in Figure 3.

**Figure 2:**
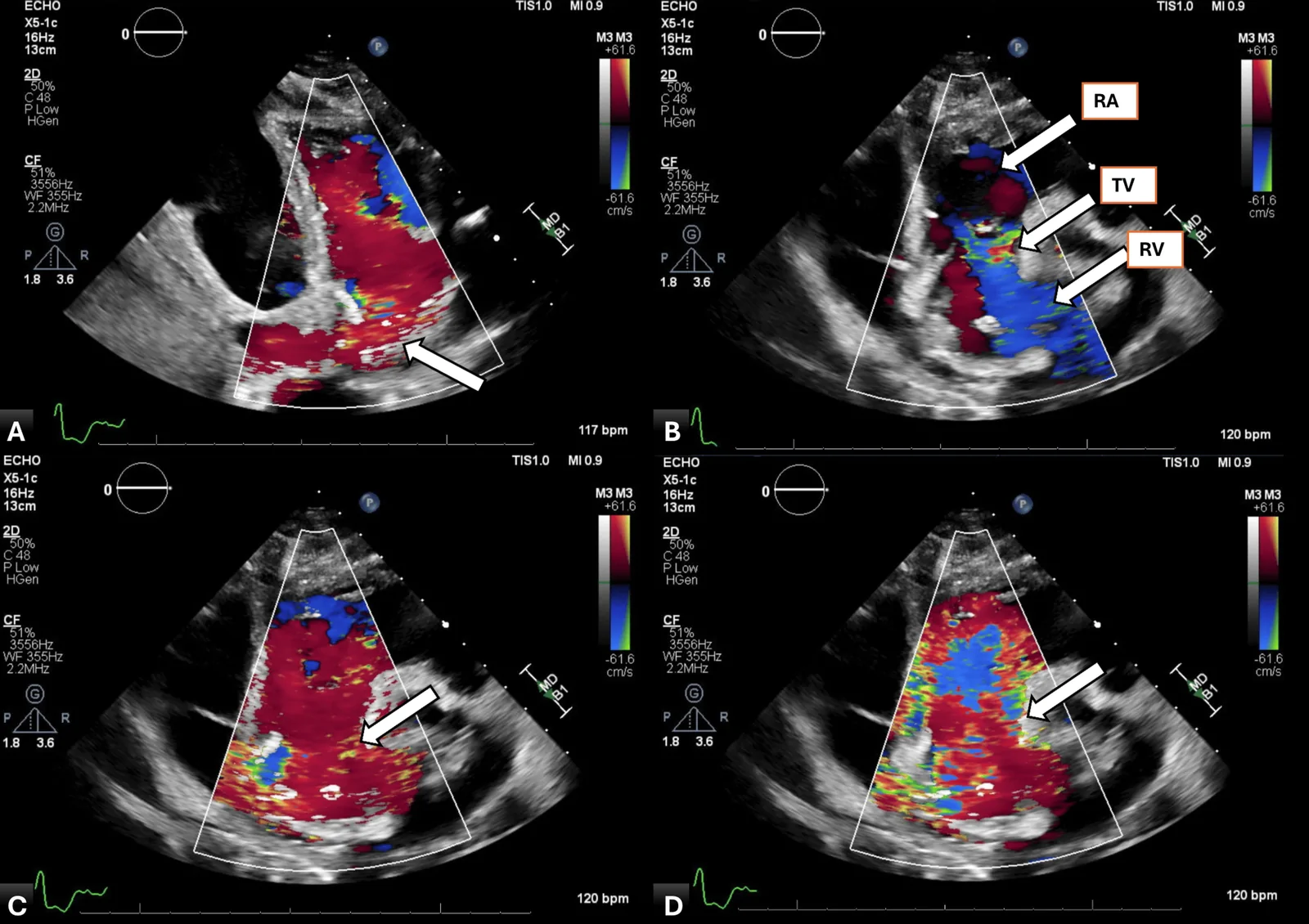
Transthoracic color Doppler demonstration of moderate-to-severe tricuspid regurgitation during acute hemodynamic deterioration. (A-D) Representative color Doppler echocardiographic views obtained during persistent sinus tachycardia (117-120 beats/min) demonstrate a broad, turbulent mosaic regurgitant jet traversing the tricuspid valve from the right ventricle into the right atrium, consistent with moderate-to-severe tricuspid regurgitation. Arrows highlight the abnormal flow across the tricuspid valve. Panel B identifies the RV, TV, and RA, arranged from the near field to the far field. These findings were interpreted in conjunction with mildly to moderately reduced right ventricular systolic function and a large loculated pericardial effusion producing regional right-sided chamber compression. Abbreviations: RA, right atrium; RV, right ventricle; TV, tricuspid valve.

**Figure 3:**
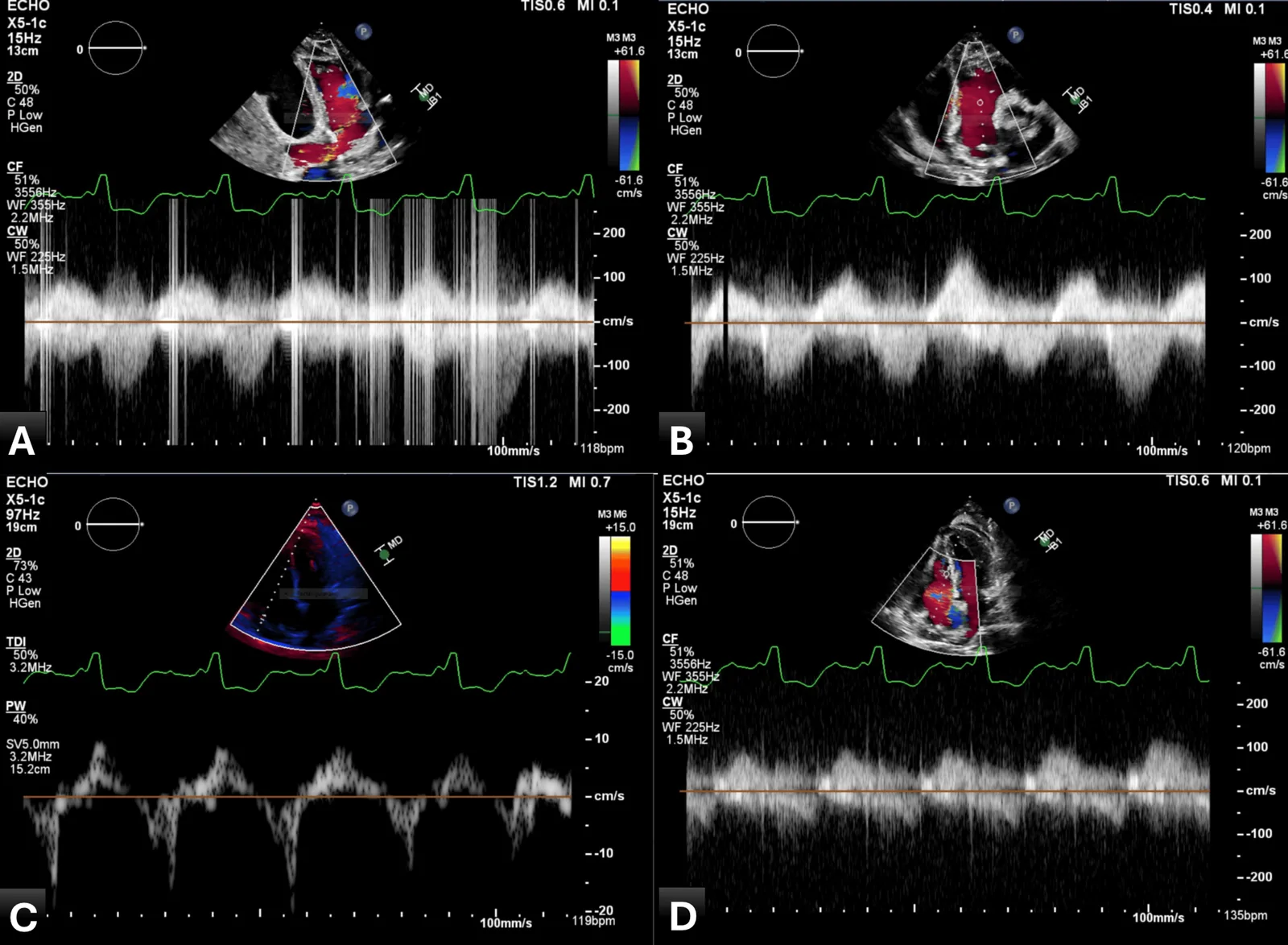
Doppler echocardiographic assessment during acute hemodynamic deterioration. (A, B, D) Color-guided continuous-wave Doppler recordings obtained from apical views at heart rates of 118-135 beats/min demonstrate dynamic transvalvular flow profiles. (C) Pulsed-wave tissue Doppler imaging demonstrates atrioventricular annular velocities at a heart rate of 119 beats/min. Evaluation of the complete echocardiographic study documented respiratory variation of approximately 23% across the mitral inflow and 35% across the tricuspid inflow. These Doppler findings were interpreted together with the large anterior loculated pericardial collection, right ventricular compression, and inferior vena cava plethora rather than as isolated evidence of tamponade.

Intraoperative transesophageal echocardiography subsequently demonstrated a small, hyperdynamic left ventricle with an estimated ejection fraction of 70%-75% and normal right ventricular size and systolic function. The bioprosthetic aortic valve was well seated, with normally mobile leaflets, an acceptable transvalvular gradient, and no visible vegetation. A complex organizing pericardial collection containing both loculated fluid and semisolid material was identified, including an extrinsic semisolid component impinging on the superolateral aspect of the right atrium. Unlike the transthoracic study, the transesophageal examination did not show classic global echocardiographic tamponade physiology. Following surgical drainage, cardiac filling improved and the liquid components were substantially evacuated, although some organized semisolid material persisted.

Multidisciplinary board discussion

The patient’s rapidly evolving clinical picture was evaluated jointly by the critical care, cardiology, cardiothoracic surgery, cardiac anesthesiology, and nephrology teams. The differential diagnosis included dialysis-associated intravascular depletion, recurrent infection with distributive shock, primary ventricular dysfunction, and obstructive shock caused by the recurrent postoperative pericardial collection. The absence of fever, leukocytosis, bacteremia, or prosthetic-valve vegetation made uncontrolled infection less likely as the principal cause of his acute deterioration. Conversely, progressive hypotension requiring norepinephrine, persistent tachycardia, regional right-sided chamber compression, inferior vena cava plethora, and the large organized pericardial collection supported a clinical diagnosis of regional cardiac tamponade with obstructive physiology.

The discordance between transthoracic and intraoperative transesophageal echocardiography was interpreted in the context of the focal postoperative distribution and semisolid composition of the collection. The patient’s severe anasarca, abnormal intravascular and extravascular volume distribution, ongoing dialysis requirements, recent cardiothoracic operations, and postoperative adhesions further complicated interpretation of conventional examination and echocardiographic criteria.

The decision to proceed with drainage was therefore based on the integrated clinical and hemodynamic picture rather than on any single ultrasonographic finding. Because untreated mechanical cardiac compression in a deteriorating patient could be fatal and the organized collection was unlikely to be adequately treated by percutaneous drainage, the multidisciplinary team recommended urgent surgical decompression. Aggressive ultrafiltration was temporarily limited until the suspected obstructive process could be addressed, with a plan to institute continuous renal replacement therapy afterward for controlled volume removal.

Diagnosis and management

The clinical diagnosis was recurrent, loculated postoperative pericardial effusion causing regional right-sided cardiac compression and obstructive physiology, superimposed on severe systemic volume overload, complex bilateral pleural disease, and acute hypoxemic respiratory failure. Although classic circumferential tamponade was not demonstrated on intraoperative transesophageal echocardiography, the absence of global echocardiographic criteria did not exclude clinically significant regional tamponade.

On Day 41, the patient underwent an urgent right mini-thoracotomy and repeat surgical pericardial window. Dense pleural adhesions were encountered, and the pericardium was opened inferiorly toward the level of the inferior vena cava. A large serous collection was evacuated from the pericardial and right pleural spaces, with approximately 680 mL drained in total. A Blake drain was left in the right pleural space. No operative complications were reported, and the patient was extubated and transferred to the cardiovascular intensive care unit in stable condition. Post-drainage transesophageal imaging demonstrated improved cardiac filling and substantial evacuation of the liquid components, although some organized semisolid material persisted.

Norepinephrine was discontinued following postoperative hemodynamic stabilization. Continuous venovenous hemodialysis was initiated to permit controlled removal of the profound excess volume in the setting of recent shock. After hemodynamic stabilization and substantial decongestion, continuous therapy was discontinued, and intermittent hemodialysis was resumed. His antimicrobial course for prior *S. lugdunensis* endocarditis was completed with cefazolin, daptomycin, and rifabutin under the direction of the infectious disease service. Persistent sinus tachycardia was managed with metoprolol, with dosing adjusted around dialysis and hypotension, while midodrine was used when necessary to support blood pressure.

Clinical outcome

Following surgical decompression and controlled volume removal, the patient’s hemodynamic and respiratory status improved. Vasopressor support was discontinued, oxygen requirements diminished, and he was ultimately comfortable on room air. His anasarca and pulmonary edema substantially resolved, and his heart rate gradually decreased from 140-150 beats/min to approximately 70-100 beats/min. Serial chest imaging demonstrated improvement in the bilateral pleural effusions and adjacent lower-lung opacities without pneumothorax. The surgical incisions remained clean and intact, and the thoracic drains were removed as their output decreased.

The temporal improvement in cardiac filling, blood pressure, vasopressor requirement, oxygenation, and tachycardia following surgical drainage supported the presence of a clinically meaningful obstructive component, despite the absence of uniform classic echocardiographic findings.

By the end of the hospitalization, the patient was afebrile, alert, ambulatory with assistance, free of chest pain or dyspnea, and maintaining normal oxygen saturation on room air. His hemoglobin had improved to approximately 9.8 g/dL, and his platelet count to 235 × 10⁹/L. At discharge on Day 54, his heart rate was 76 beats/min, blood pressure was 117/66 mmHg, respiratory rate was 20 breaths/min, and oxygen saturation was 100% on room air. He was discharged home after hemodialysis with continuation of his thrice-weekly outpatient dialysis schedule and planned cardiothoracic surgery follow-up with interval chest radiography and laboratory testing. No recurrent hemodynamic compromise attributable to the pericardial collection was documented before discharge.

## Discussion

Background 

Postpericardiotomy syndrome was first recognized in 1952 following mitral commissurotomy and is now classified among the post-cardiac injury syndromes resulting from myocardial or pericardial trauma [6]. Postoperative pericardial effusion is a broader entity: early collections may result from surgical bleeding or impaired drainage, whereas later collections may reflect pericardial inflammation and be accompanied by pleural involvement [6,7]. A systematic review of 26 studies involving 8,495 patients reported a pooled incidence of postoperative pericardial effusion of 36%, although only approximately 3% of patients required reintervention [1].

Cardiac tamponade is not an ultrasonographic diagnosis but a clinical and hemodynamic syndrome in which elevated intrapericardial pressure impairs cardiac filling and compromises cardiac output [7]. Echocardiography is essential for identifying pericardial fluid and its physiologic consequences; however, no individual echocardiographic finding independently establishes or excludes tamponade [2,3,7,8]. This distinction is especially important following cardiac surgery, when adhesions and loculations may produce focal compression of an individual chamber rather than the uniform pressure transmission associated with circumferential tamponade [2,3,9]. Consequently, regional tamponade may present with variable symptoms and without classic examination or echocardiographic findings [2,9].

Dialysis dependence further complicates interpretation because pericardial disease, severe systemic congestion, and abnormal intravascular volume distribution may coexist [10,11]. The present case is noteworthy because clinically consequential regional compression occurred in a young dialysis-dependent patient despite profound total-body volume overload, recurred after a previous pericardial window, and produced incomplete and apparently discordant findings across transthoracic echocardiography, CT, and intraoperative transesophageal echocardiography.

Pathology and pathophysiology 

Postoperative pericardial effusion is an acquired process initiated by injury to the pericardial mesothelial and submesothelial tissues. Surgical trauma, inflammation, and microvascular bleeding introduce serous fluid, erythrocytes, fibrin, and inflammatory mediators into the pericardial space [2,6,9]. Fibrin deposition, organization of blood products, and postoperative adhesions may subsequently partition the pericardial space into noncommunicating locules [2,6,9]. A previous pericardial window may therefore fail to drain a newly isolated compartment.

When pressure within a locule exceeds the filling pressure of the adjacent cardiac chamber, focal compression and impaired diastolic filling occur [2,9]. The right atrium and right ventricle are particularly vulnerable because of their relatively low intracavitary pressures [2,9]. Reduced venous return and stroke volume may then produce compensatory tachycardia, followed by hypotension and obstructive shock. Because pressure is not transmitted uniformly, jugular venous distension, pulsus paradoxus, or global chamber collapse may be absent, and different imaging modalities may demonstrate apparently discordant findings [2,3,9].

Profound edema and ascites do not necessarily indicate adequate effective cardiac filling, particularly in dialysis-dependent patients with abnormal fluid distribution [10,11]. In this case, the operative findings of serous fluid and semisolid organized material supported a mixed postoperative inflammatory and hemorrhagic process. However, the absence of complete fluid characterization and pericardial histopathology prevents attribution to a single mechanism.

Comparative analysis and literature review 

Most postoperative pericardial effusions are asymptomatic or produce nonspecific dyspnea and chest discomfort, while progression to clinically significant tamponade remains uncommon [1]. Regional postoperative tamponade may instead present with unexplained tachycardia, hypotension, oliguria, respiratory deterioration, or increasing vasopressor requirements without the complete classic triad of hypotension, jugular venous distension, and muffled heart sounds [2,3,9]. The diagnosis must therefore be based on the overall clinical and hemodynamic trajectory, with imaging used to identify and anatomically characterize the responsible collection rather than to establish the diagnosis in isolation [2,3,7].

Gopalakrishnan and Pillai described a postoperative patient with a localized anterior pericardial effusion and right-ventricular diastolic free-wall collapse whose symptoms and wall motion abnormalities improved after urgent drainage of 700 mL of pericardial fluid [5]. Adi et al. reported posterior loculated pericardial clots after coronary artery bypass grafting that were difficult to identify using transthoracic echocardiography but were recognized using transesophageal echocardiography [2]. Hamdan et al. described a 75-year-old woman with a loculated postoperative pericardial hematoma causing right-atrial compression and hemodynamic deterioration following aortic-valve replacement [9]. The present case shared the regional right-sided compression and improvement after decompression described in these reports but differed because of the patient’s young age, dialysis dependence, recurrent effusion after a previous surgical window, severe systemic volume overload, complex bilateral pleural collections, and simultaneous liquid and semisolid pericardial components.

Transthoracic echocardiography remains the preferred initial imaging modality because it can evaluate effusion size, chamber compression, respiratory inflow variation, ventricular function, and inferior vena cava behavior at the bedside [7]. Nevertheless, these findings must be interpreted within the clinical context. In a cohort of 195 patients with pericardial effusion, hypotension, increasing effusion size, right-ventricular diastolic collapse, and marked mitral inflow variation were associated with the need for urgent drainage, but no single finding was determinative [8]. In the present patient, hypotension, persistent tachycardia, increasing vasopressor requirements, a large regional collection, right-ventricular compression, and inferior vena cava plethora collectively supported obstructive physiology. The transthoracic examination therefore provided supportive evidence for the clinical diagnosis rather than independently diagnosing tamponade.

CT complemented echocardiography by defining the size, location, and loculated configuration of the pericardial collection. Intraoperative transesophageal echocardiography did not demonstrate classic global tamponade physiology but identified an organized semisolid component impinging on the right atrium. This apparent discordance did not exclude clinically important regional compression. Loculated postoperative collections do not transmit pressure uniformly, and the affected chamber may vary according to the location of the collection, imaging plane, loading conditions, and composition of the accumulated material [2,3,9]. Consequently, the absence of conventional global tamponade findings should not provide reassurance when the patient’s clinical condition and anatomic imaging demonstrate progressive regional cardiac compression.

The differential diagnosis included distributive shock from recurrent infection, primary cardiogenic shock, dialysis-related intravascular depletion, and severe systemic congestion. The absence of fever, leukocytosis, positive cultures, or a new prosthetic-valve abnormality made uncontrolled infection less likely, while preserved left-ventricular systolic function argued against isolated left-sided cardiogenic shock. The decisive feature was the patient’s continued hemodynamic deterioration in association with a large regional postoperative collection. Untreated hemodynamically significant tamponade may progress to refractory obstructive shock and death; therefore, urgent decompression is indicated when clinical compromise is present [7].

Echocardiography-guided pericardiocentesis is generally appropriate for accessible, free-flowing collections, and CT guidance may facilitate drainage of selected loculated effusions [12]. However, organized or semisolid material constrained by dense postoperative adhesions may not be adequately evacuated percutaneously [2,9,12]. The decision to proceed with repeat mini-thoracotomy and surgical drainage was therefore based on the collection’s recurrent, loculated, and organized nature. Controlled renal replacement therapy was initiated after decompression to address the severe systemic volume overload without relying on ultrafiltration as treatment for mechanical cardiac compression.

Following evacuation of approximately 680 mL from the pericardial and right pleural spaces, cardiac filling improved, vasopressor support was discontinued, and the patient’s tachycardia and respiratory status subsequently improved. This temporal response supports a clinically meaningful obstructive component, although it does not prove that regional tamponade was the sole cause of deterioration. The absence of simultaneous invasive hemodynamic measurements, complete pericardial-fluid analysis, and long-term echocardiographic follow-up should therefore be acknowledged.

What we learned from this case 

This case highlights the diagnostic challenge of recognizing regional cardiac tamponade in a postoperative patient when classic echocardiographic findings are incomplete or discordant. Despite severe systemic volume overload with anasarca, pulmonary edema, ascites, and recurrent pleural effusions, the patient developed progressive tachycardia, hypotension, respiratory compromise, and vasopressor dependence. Transthoracic echocardiography demonstrated a large anterior pericardial collection with right ventricular compression and inferior vena cava plethora, whereas intraoperative transesophageal echocardiography did not show classic global tamponade physiology. Instead, it revealed a complex, organized collection causing focal right-sided cardiac compression.

This case demonstrates that postoperative pericardial collections may produce localized hemodynamic compromise through adhesions, loculations, and organized material without the textbook findings of circumferential tamponade. Therefore, the absence of classic echocardiographic criteria should not exclude clinically significant obstructive physiology when the patient's clinical trajectory and imaging findings suggest regional compression.

It also emphasizes that profound total-body volume overload does not necessarily reflect adequate effective cardiac filling. Temporarily limiting aggressive ultrafiltration, proceeding with surgical decompression, and subsequently using controlled renal replacement therapy allowed stabilization and decongestion. Following drainage, cardiac filling improved, vasopressor support was discontinued, and tachycardia and respiratory status improved.

Overall, regional postoperative tamponade should remain a clinical and hemodynamic diagnosis supported by multimodality imaging rather than being excluded solely by the absence of classic global echocardiographic findings.

Limitations

This report is limited by its retrospective nature and incomplete access to outside-hospital records. Resuscitation records related to the cardiac arrest 20 days before the index admission were unavailable, preventing verification of arrest downtime and resuscitation duration. During the index hospitalization, the diagnosis of regional tamponade was based on clinical deterioration and hemodynamic assessment supported by multimodality imaging. The absence of simultaneous invasive hemodynamic measurements limited quantification of the contribution of regional cardiac compression to the shock state. Transthoracic and intraoperative transesophageal examinations were also performed under different clinical conditions, limiting direct comparison.

Clinical improvement followed drainage of both the pericardial and pleural spaces and subsequent controlled volume removal; therefore, recovery cannot be attributed exclusively to pericardial decompression. The reported drainage volume of approximately 680 mL represented fluid evacuated from both spaces, and the pericardial volume alone could not be determined. Incomplete pericardial-fluid characterization and unavailable pericardial histopathology limited definitive attribution of the effusion to postoperative inflammation, bleeding, or kidney failure. Follow-up was limited to discharge on Day 54, precluding assessment of durable resolution or subsequent recurrence. These findings describe an individual clinical course and cannot establish comparative treatment effectiveness.

## Conclusions

Regional postoperative cardiac tamponade is a clinical and hemodynamic syndrome whose presentation may vary according to the location and composition of the pericardial collection and the patient’s underlying volume status. This case merits publication because recurrent regional right-sided cardiac compression developed in a young dialysis-dependent patient despite profound total-body volume overload, a previous pericardial window, and incomplete and discordant echocardiographic findings. The diagnosis was driven by progressive tachycardia, hypotension, respiratory compromise, and vasopressor dependence, while multimodality imaging supported and anatomically characterized the suspected obstructive process. The subsequent improvement in cardiac filling and hemodynamic status following surgical decompression supported a clinically meaningful regional tamponade physiology. This case demonstrates that neither severe systemic volume overload nor the absence of classic global echocardiographic findings should provide false reassurance. Prompt multidisciplinary evaluation and drainage may be lifesaving when the clinical trajectory and imaging findings indicate regional cardiac compression.
